# Human Islet Microtissues as an In Vitro and an In Vivo Model System for Diabetes

**DOI:** 10.3390/ijms22041813

**Published:** 2021-02-11

**Authors:** Joan Mir-Coll, Tilo Moede, Meike Paschen, Aparna Neelakandhan, Ismael Valladolid-Acebes, Barbara Leibiger, Adelinn Biernath, Carina Ämmälä, Ingo B. Leibiger, Burcak Yesildag, Per-Olof Berggren

**Affiliations:** 1 InSphero AG, Wagistrasse 27a, 8952 Schlieren, Switzerland; joan.mir@insphero.com (J.M.-C.); aparna.neelakandhan@insphero.com (A.N.); adelinn.biernath@insphero.com (A.B.); 2 The Rolf Luft Research Center for Diabetes and Endocrinology, Department of Molecular Medicine and Surgery, Karolinska Institutet, Karolinska Sjukhuset L1:03, 17176 Stockholm, Sweden; tilo.moede@ki.se (T.M.); paschen.meike@gmail.com (M.P.); ismael.valladolid.acebes@ki.se (I.V.-A.); ingo.leibiger@ki.se (I.B.L.); 3 Bioscience, Research and Early Development, Cardiovascular, Renal and Metabolism, BioPharmaceuticals R&D, AstraZeneca, Gothenburg, 43138 Mölndal, Sweden; carina.ammala@astrazeneca.com

**Keywords:** diabetes, β-cell dysfunction, glucotoxicity, glucolipotoxicity, pancreatic islets, viral transduction, insulin resistance, transplantation, diet-induced obesity

## Abstract

Loss of pancreatic β-cell function is a critical event in the pathophysiology of type 2 diabetes. However, studies of its underlying mechanisms as well as the discovery of novel targets and therapies have been hindered due to limitations in available experimental models. In this study we exploited the stable viability and function of standardized human islet microtissues to develop a disease-relevant, scalable, and reproducible model of β-cell dysfunction by exposing them to long-term glucotoxicity and glucolipotoxicity. Moreover, by establishing a method for highly-efficient and homogeneous viral transduction, we were able to monitor the loss of functional β-cell mass in vivo by transplanting reporter human islet microtissues into the anterior chamber of the eye of immune-deficient mice exposed to a diabetogenic diet for 12 weeks. This newly developed in vitro model as well as the described in vivo methodology represent a new set of tools that will facilitate the study of β-cell failure in type 2 diabetes and would accelerate the discovery of novel therapeutic agents.

## 1. Introduction

Type 2 diabetes mellitus (T2D) is a metabolic disease characterized by hyperglycemia as a result of impaired insulin secretion and action, estimated to affect 422 million people worldwide [1]. The two hallmarks of the disease are insulin resistance in peripheral tissues, tightly associated with increased body mass index (BMI), and dysfunction of insulin-producing β-cells found in pancreatic islets. When β-cell failure occurs, these cells cannot compensate for increased insulin demand and cannot keep blood glucose levels within a normal range [2]. The chronic exposure of β-cells to elevated glucose concentrations (glucotoxicity) as well as the combination of elevated concentrations of glucose and lipids (glucolipotoxicity), are thought to be the main factors responsible for the decline of β-cell function in T2D [3,4].

Numerous in vivo and in vitro models have been developed to study T2D relevant β-cell dysfunction [5]. Rodent models of T2D have provided valuable insights into the regulation of β-cell function and its adaptation to pathological conditions [6]. However, it is clear that rodent β-cells differ from human β-cells in parameters such as response to different stressors, proliferative capacity under insulin resistance, glucose uptake, kinetics of insulin secretion, cellular composition and architectural distribution, and transcriptional profile [7]. Various rodent and human cell lines (as well as stem-cell-derived β-like cells) have been used in vitro, but they also present certain limitations such as lack of 3D structure, altered intercellular communication, absent paracrine interactions, and altered proliferation and stress response due to immortalization [8]. Primary human islets are thus considered to be the best available tool for the study of β-cell function and failure in T2D. However, their experimental use is hindered by heterogeneity in islet size and cellular composition, variable degrees of exocrine tissue contamination (low purity), batch-to-batch differences in functionality [9], and short ex vivo lifespan due in part to necrosis in the core of large islets [10]. Previously, we have described how these challenges can be overcome with the use of standardized human islet microtissues, produced by enzymatic dissociation and controlled scaffold-free hanging-drop-based reaggregation of primary islet cells [11]. Islet microtissues are uniform in size and cellular composition, and display long-term and stable functionality and viability during in vitro culture, thus could enable disease-modeling under T2D-relevant conditions. 

Another historical challenge in studies with primary human islets has been their limited amenability to genetic manipulation. Viral transduction techniques, among the most frequently used methods to induce changes in gene expression, have delivered little success with intact islets as the transduction remains mostly restricted to cells in the periphery of the islets, due to limited accessibility of viral vectors to the inner cells of the 3D structure. Recently, several authors have reported methods for highly efficient viral transduction, including transduction of dissociated islet cells in suspension before pseudoislet formation [12] or by using a combination of microfluidic flow and transient tissue expansion [13]. Based on this, the production process of human islet microtissues theoretically opens the possibility to achieve highly efficient genetic manipulation by transducing islet cells while they are dispersed. Thus, islet microtissues are a standardized, functionally robust, and long-lived in vitro human islet model, that can be amenable to genetic manipulation and exploited in many applications for the study of β-cell dysfunction in T2D.

In a previous study we investigated β-cell dysfunction and β-cell insulin resistance by in vivo monitoring of reporter mouse islets transplanted into the anterior chamber of the eye (ACE) of mice exposed to a high-fat-high-sucrose diet (HFHSD) [14]. Here, following the same approach, we aimed to study the dysfunction of human β-cells by measuring the functional β-cell mass in transduced islet microtissues transplanted into the ACE of diet-induced obese/diabetic mice. To achieve this goal we (1) demonstrated that human islet microtissues are suitable to study glucotoxicity/glucolipotoxicity-induced β-cell dysfunction, (2) established the ideal method for highly efficient viral transduction of islet microtissues, (3) ensured that islet microtissues survive, engraft, and maintain their functionality following transplantation into the ACE of immune-deficient mice, and (4) assessed changes in functional β-cell mass following HFHSD by quantification of glucose-responsive fluorescent reporters introduced to human islet microtissues prior to transplantation.

## 2. Results

### 2.1. Modeling β-Cell Dysfunction in Human Islet Microtissues with Glucotoxicity and Glucolipotoxicity In Vitro

**Figure 1:**
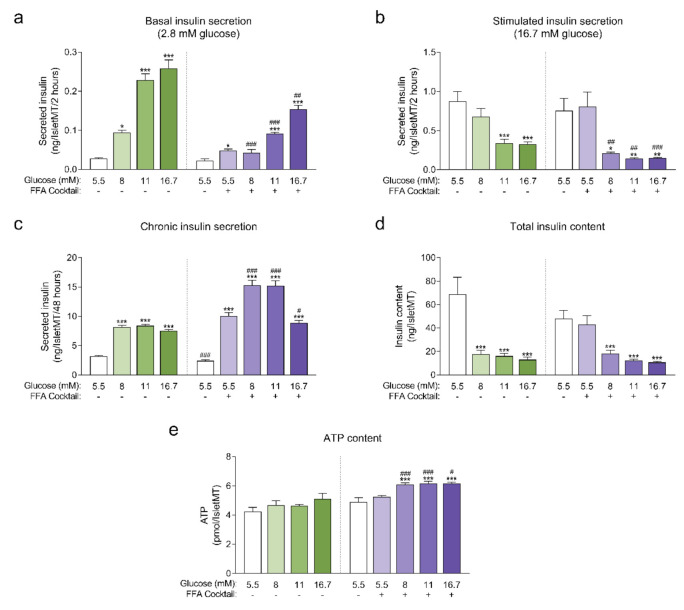
(**a**) Basal insulin secretion at 2.8 mM glucose at the end of 14-day culture of human islet microtissues (IsletMT) in media containing the indicated glucose concentrations and a free fatty acid (FFA) cocktail (*n* = 5–6); (**b**) Stimulated insulin secretion at 16.7 mM glucose at the end of 14 days of incubation (*n* = 5–6); (**c**) Chronic insulin secretion to the culture media corresponding to the last 48 h of incubation (*n* = 10–12); (**d**) Intracellular insulin content of islet microtissues after 14 days of incubation (*n* = 11–12). (**e**) Intracellular ATP content of islet microtissues after 14 days of incubation (*n* = 9–12). Outliers were detected with ROUT’s test (Q = 5%). Statistical analysis was performed with one-way ANOVA of glucotoxicity and glucolipotoxicity groups separately. Dunnett’s multiple comparisons tests against the 5.5 mM glucose control of each condition are shown: *** *p* < 0.001, ** *p* < 0.01, * *p* < 0.5. Unpaired Student’s *t*-test was performed to compare each glucose concentration with and without FFAs: ### *p* < 0.001, ## *p* < 0.01, # *p* < 0.5.

Human islet microtissues produced from one individual healthy donor (UNOS ID# AEBB007, 59 years old, male, BMI 26.89, HbA1c 5.8%) were exposed to increasing concentrations of glucose in the culture medium, with or without the addition of a mix of saturated and unsaturated free fatty acids (FFAs, 200 µM oleate, 100 µM palmitate) for 14 days. The insulin secretory function at basal (2.8 mM) and stimulatory (16.7 mM) glucose concentrations were assessed by a 2-hour static incubation at the end of the 14-day culture. Basal insulin secretion was increased in islet microtissues chronically exposed to high glucose and high FFA concentrations alone or in combination. The increase in basal insulin secretion correlated with increasing glucose concentrations in the culture medium and was slightly higher for conditions without FFAs for the respective glucose concentrations (Figure 1a). Basal insulin secretion was also slightly increased by FFAs alone. Dysregulated insulin secretion at non-stimulatory glucose concentrations is typically regarded as a sign of β-cell immaturity and/or dysfunction [15,16,17,18]. Likewise, a dysfunction in glucose-stimulated insulin secretion (GSIS) was observed in islet microtissues exposed to glucotoxicity and glucolipotoxicity (Figure 1b), one of the hallmarks of β-cell failure in T2D. Glucose alone impaired GSIS starting at 11 mM. FFAs alone did not influence GSIS, but did contribute to β-cell dysfunction at the respective glucose concentrations. As a result, addition of FFAs resulted in a significantly reduced GSIS starting already at 8 mM glucose. After exposure to 16.7 mM glucose with or without FFAs, the increase in basal insulin secretion and the decrease in GSIS was so drastic that the islets secreted the same levels of insulin under both basal and stimulatory conditions. As expected, exposure to high levels of glucose and FFAs led to an increase in the insulin secretory demand, which was evident by the higher levels of insulin that accumulated in the conditioned culture medium (Figure 1c). Moreover, FFAs alone also led to an increase in chronic insulin release. 

Unlike the glucose-dependent changes in basal and stimulated insulin secretion, the increase in chronic insulin secretion seemed to reach a plateau at 8 mM glucose and was not further increased at 11 mM and 16.7 mM glucose. Interestingly, a drop in chronic insulin secretion was observed with glucolipotoxicity at 16.7 mM glucose compared to 8 mM and 11 mM glucose, which suggests that under these conditions β-cells were not able to keep up with the increased insulin secretory demand and reached the “breaking point” known as β-cell failure [2]. This decline in chronic insulin secretion was not observed in the absence of FFAs, supporting the idea that the combined toxicity of high glucose and high lipids is required for β-cell failure to occur. Additionally, β-cells exhibited a great reduction in intracellular insulin content following exposure to glucotoxic and glucolipotoxic culture conditions (Figure 1d). Insulin content was significantly decreased even for conditions where GSIS was not affected. These results were reproduced with glucolipotoxicity in a second islet donor (Appendix A).

Next, we quantified intracellular ATP content following the 14-day incubation period (Figure 1e), as a decline in ATP content may indicate a decrease in islet viability or metabolic activity. We observed no statistically significant differences in ATP content of islet microtissues cultured under glucotoxic conditions compared to healthy controls. On the other hand, levels of ATP detected in islet microtissues cultured under glucolipotoxicity were significantly higher than levels in healthy controls. Despite the increase in islet microtissue ATP levels, no significant changes were detected in cellular proliferation rates or total cell numbers (Appendix B). Glucolipotoxic culture conditions also did not alter the number of cleaved caspase 3 positive cells and total caspase 3/7 activity in the islet microtissues (Appendix B). We concluded that the differences in insulin secretion, intracellular insulin, and ATP content under glucolipotoxic stress did not correlate with changes in cellular viability.

### 2.2. Establishment of the Ideal Method for Highly Efficient Viral Transduction of Human Islet Microtissues

Once we proved the feasibility of studying T2D relevant β-cell dysfunction in human islet microtissues in vitro, we decided to undertake a similar approach in an in vivo setting. For this purpose, we decided to transduce human islet microtissues with βFLUOMETRI, a fluorescent biosensor that allows real time monitoring of β-cell function in response to glucose stimulation [14], and to transplant the transduced islet microtissues into the ACE of immune-deficient mice.

**Figure 2:**
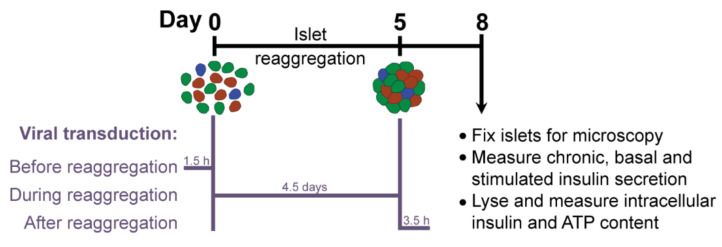
Experimental outline.

βFLUOMETRI is an adenoviral vector that combines three expression cassettes in a single construct: the DsRed2 gene driven by a fragment of the rat insulin-1 gene promoter (−410/+1 bp; RIP1-410), the enhanced green fluorescent protein (EGFP) gene driven by a fragment of the rat β-cell–active glucokinase gene promoter (−278/+123 bp; bGK-278) and the Cerulean Fluorescent Protein (CFP) gene driven by the cytomegalovirus (CMV) promoter. While RIP1-410 and bGK-278 contain metabolic response elements for glucose, insulin, Ca^2+^, and cAMP, the CMV promoter does not respond to these metabolic stimuli and serves as a reference [14]. First, we optimized the transduction protocol to obtain efficiently and homogeneously transduced islet microtissues with intact β-cell function. Taking advantage of the dissociation stage in the production of islet microtissues, during which viral particles have better access to single islet cells, we compared the efficiency of transducing the islets with a range of multiplicities of infection (MOI) at three different stages (Figure 2): before, during, and after microtissue reaggregation. Additionally, we quantified the expression of the fluorescent reporters in predefined concentrical regions of the islets and assessed the effects of viral transduction on β-cell functionality.

#### 2.2.1. Quantification of Viral Transduction Efficiency

**Figure 3:**
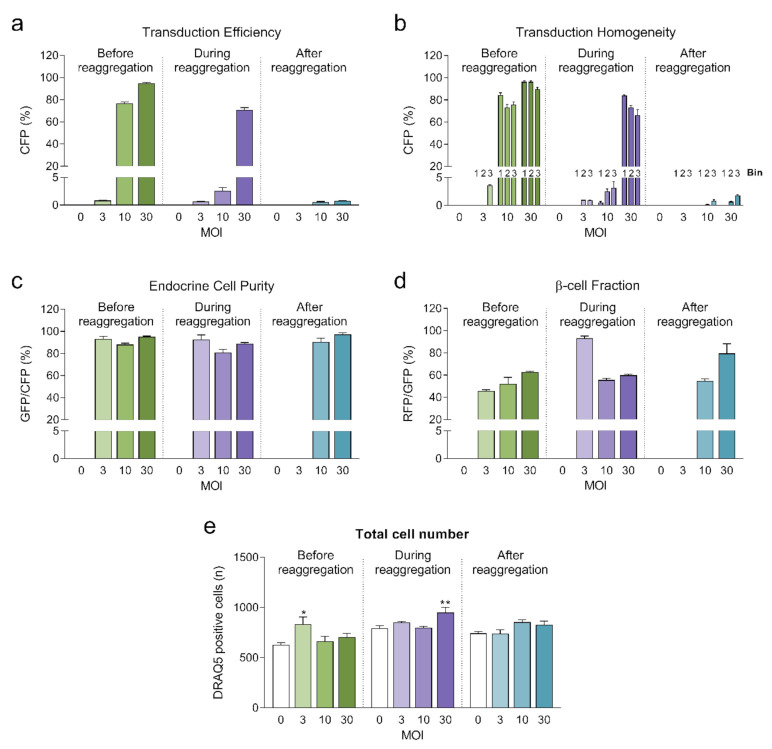
(**a**) Transduction efficiency calculated as the percentage of cells expressing Cerulean Fluorescent Protein (CFP) (*n* = 5–6). Statistical analysis was performed with one-way ANOVA of each transduction method separately. Dunnett’s multiple comparisons tests against the untransduced control of each condition are shown: * *p* < 0.05, ** *p* < 0.01; (**b**) Homogeneity of transduction shown as the transduction efficiency in 3 predefined regions: bin 1 represents the core, bin 2 represents the middle region and bin 3 represents the periphery; (**c**) Endocrine cell purity calculated as the percentage of CFP positive cells expressing enhanced green fluorescent protein (EGFP). (**d**) β-cell fraction calculated as the percentage of EGFP positive cells expressing DsRed2. (**e**) Total number of cells analyzed represented as DRAQ5 positive nuclei count. Outliers were detected with ROUT’s test (Q = 5%).

Highest transduction efficiencies, defined by the percentage of cells expressing CFP, were achieved by transducing dissociated islet cells prior to reaggregation (UNOS ID# AEL5345, 48 years old, male, BMI 32.38, HbA1c 5.6%) (Figure 3a. Example confocal microscopy images are shown in Appendix C). At MOI 30 and MOI 10, 94.8% and 76.6% of the cells, respectively, expressed CFP in the newly formed islet microtissues. Transduction of islet cells during the reaggregation stage in the hanging drops resulted in 70.9% of the cells being transduced at MOI 30. It is important to note that, for the same MOI, the concentration of viral particles was 5-fold higher for the transduction protocol before aggregation compared to the protocol during aggregation, which may explain the lower transduction efficiency despite the increased viral exposure time. In the conditions with transduction efficiency >70%, we observed that CFP positive cells were homogeneously distributed across all depths of the islet microtissues (Figure 3b). On the other hand, transduction of the islet microtissues after reaggregation resulted in very low efficiency (0.78% at MOI 30) and a tendency for the transduction of cells in the islet periphery. This transduction efficiency was lower than what is normally obtained when transducing whole islets, which may be explained by the low concentration of viral particles, which was 88% lower in this protocol compared to transduction before reaggregation for the same MOI as a result of low cell to medium ratio.

In addition, the three fluorescent reporters encoded in βFLUOMETRI, driven by different promoters, enabled us to assess the cellular composition of the islet microtissues following viral transduction. Endocrine cells (displaying EGFP expression) represented 80–95% of all transduced cells expressing CFP (Figure 3c), consistent with the fact that the majority of the cells in the islet microtissues are endocrine cells. In the same way and considering only the conditions in which high efficiency was achieved, β-cells (displaying DsRed2 expression) represented 52–62% of all endocrine cells expressing EGFP (Figure 3d), in line with the expected ratio of β-cells in human pancreatic islets [19,20].

#### 2.2.2. Assessment of Islet Microtissue Functionality Following Viral Transduction

**Figure 4:**
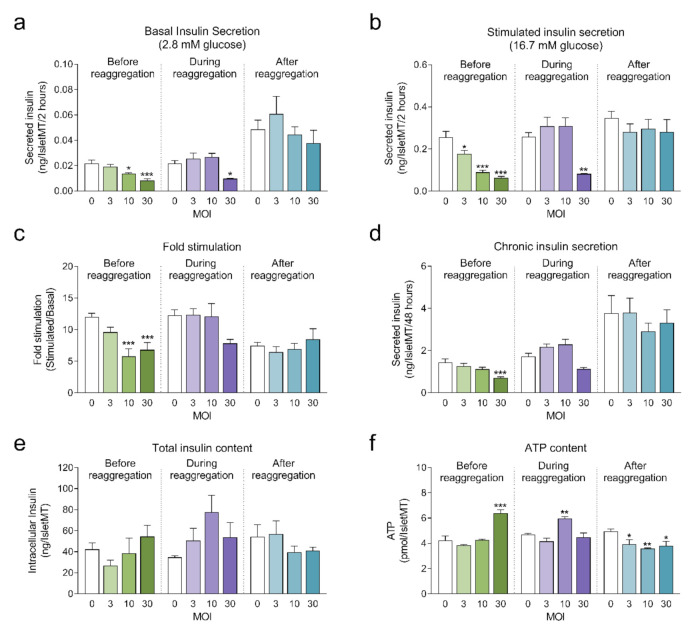
(**a**) Basal insulin secretion at 2.8 mM glucose (*n* = 5–6); (**b**) Stimulated insulin secretion at 16.7 mM glucose (*n* = 5–6); (**c**) Fold stimulation of insulin secretion calculated as the ratio between stimulated and basal secretion (*n* = 5–6); (**d**) Chronic insulin secretion to the culture media corresponding to the last 72 h in culture (*n* = 5–6); (**e**) Intracellular insulin content of islet microtissues (*n* = 5–6); (**f**) Intracellular ATP content of islet microtissues (*n* = 5–6). Outliers were detected with ROUT’s test (Q = 5%). Statistical analysis was performed with one-way ANOVA for each transduction method separately. Dunnett’s multiple comparisons tests against the untransduced control of each condition are shown: *** *p* < 0.001, ** *p* < 0.01, * *p* < 0.5.

Next, we investigated the impact of adenoviral transduction on islet viability and function. We performed a 2-hour static incubation insulin secretion assay 8 days after initiation of the reaggregation. Both basal and stimulated insulin secretion were reduced under conditions where the highest transduction efficiency was achieved (before reaggregation at MOI 10 and MOI 30, and during reaggregation at MOI 30) compared to untransduced islet microtissues (Figure 4a,b). Similarly, chronic insulin secretion to the culture media was reduced in islet microtissues transduced before and during reaggregation at MOI 30 (Figure 4d). Despite these alterations, β-cell glucose responsiveness was well preserved, with a fold stimulation of insulin secretion greater than 6.7 in all groups (Figure 4c). Importantly, adenoviral transduction of islet microtissues with high efficiency had no negative influence on intracellular insulin (Figure 4e) and ATP contents (Figure 4f). Higher ATP content was observed in islets transduced before reaggregation at MOI 30 as well as in islets transduced during reaggregation at MOI 10. Interestingly, a similar pattern, although without statistically significant differences, is observed for the total insulin content. 3D microscopic quantification revealed similar number of cells in conditions with altered ATP content (Figure 3e), therefore these differences are unlikely to be a result of altered cellular viability and most likely be due to alterations in ATP synthesis, consumption, or secretion.

Considering a balance between technical amenability, transduction efficiency, β-cell functionality and islet viability, transduction before reaggregation at MOI 10 was selected for the transplantation experiments.

### 2.3. Validation of Transplantation and Engraftment of Human Islet Microtissues in the ACE Model in NSG Mice

**Figure 5:**
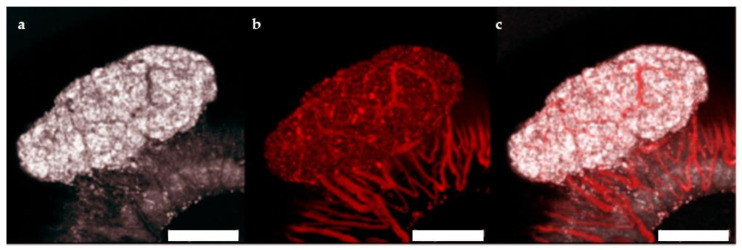
Vascularization of human islet microtissues. Maximum projection of a stack of confocal images of a human islet microtissue engrafted on the iris of a NSG mouse 8 weeks after transplantation. (**a**) Reflection image; (**b**) TRITC-dextran fluorescence; (**c**) Overlay of reflection and fluorescent images; (**a**–**c**) Scale bar is 200 µm.

The ACE represents a highly effective site for islet transplantation where the engrafted islets can be easily monitored with non-invasive imaging for gross morphology and vascularization [21,22]. To study the feasibility of transplanting human islet microtissues into the ACE of mice, 3 immune-deficient NSG mice were monitored long-term by non-invasive in vivo optical imaging following transplantation with non-transduced human islet microtissues from one healthy donor (UNOS ID# AEFT450, age 57 years old, female, Asian/Filipino, BMI 25.82, HbA1c 5.3%) into the ACE of the left eye and βFLUOMETRI-biosensor-transduced human islet microtissues from the same donor into the ACE of the right eye. Vascularization of non-transduced human islet microtissues was visualized by injection of TRITC-labelled dextran 4 and 8 weeks after transplantation (Figure 5). Islet microtissues were engrafted and vascularized with a success rate comparable to that of mouse islets. However, the vascularization of human islet microtissues took longer than that of mouse islets transplanted into the ACE of littermates. Human islet microtissue vascularization after 8 weeks (Figure 5) was comparable to vascularization of mouse islets transplanted into the ACE after 4 weeks [21,22].

**Figure 6:**
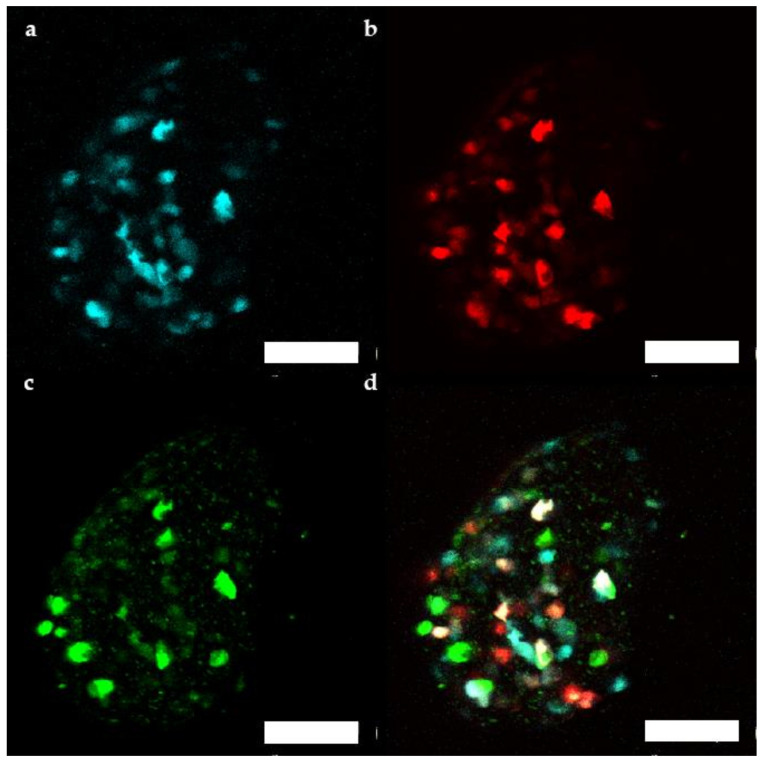
Representative maximum projection of a stack of confocal fluorescence images of a human islet microtissue expressing βFLUOMETRI obtained 3 months after transplantation into the anterior chamber of the eye (ACE) of a NSG mouse. (**a**) CMV.CFP-fluorescence; (**b**) RIP1.DsRed2-fluorescence; (**c**) bGK.EGFP-fluorescence; (**d**) Overlay of (**a**–**c**); (**a**–**d**) Scale bar is 100 µm.

Additionally, fluorescence of the transduced human microtissue grafts was imaged in vivo from 1 month up to 4 months after transplantation [22,23]. Between 39 and 93 cells/microtissue could be followed over the 3 months period. βFLUOMETRI fluorescence of transduced human islet microtissues (Figure 6) stayed detectable for several months, similar to what has been observed in mouse islets.

### 2.4. Monitoring Glucose Responsiveness In Vivo Using Reporter Islet Microtissues Transplanted into the ACE of NSG Mice

βFLUOMETRI fluorescence of the human islet microtissue grafts was imaged in vivo with non-invasive imaging using a laser scanning confocal microscope. The mice were anesthetized and human islet microtissue grafts were imaged as 3D-stacks with 3 µm step-size before glucose stimulation. After this initial imaging, mice were allowed to recover from anesthesia and an intraperitoneal glucose tolerance test (IPGTT) was performed. At 4 h post glucose injection, a second imaging was performed using the same setting as described above to obtain the 4 h-image set.

**Figure 7:**
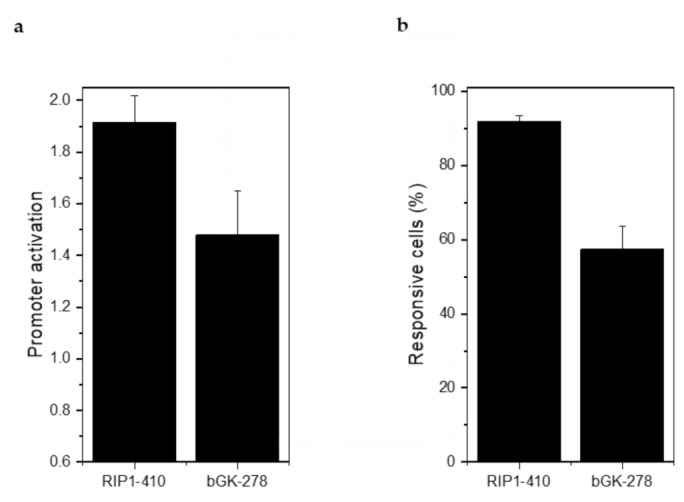
βFLUOMETRI-transduced human islet microtissues engrafted onto the iris responded to glucose stimulation by activation of the RIP1 and bGK promoters. (**a**) Activation of RIP1-410 and bGK-278 promoters after glucose injection into NSG mice in vivo 3 months after transplantation into the ACE (*n* = 3 animals and at least 3 organoids/animal were imaged). Data are expressed as mean ± SEM; (**b**) Percentage of glucose-responsive cells indicated by RIP1-410 and bGK-278 promoters of βFLUOMETRI after glucose injection into NSG mice in vivo 3 months after transplantation into the ACE (*n* = 3 and at least 3 organoids/animal were imaged).

Injection of 2 g/kg glucose led to a 1.91 ± 0.11-fold stimulation of RIP1-DsRed2 expression and a 1.48 ± 0.17-fold stimulation of bGK-EGFP expression (Figure 7a). 91.80 ± 1.70% of the β-cells responded with an increase in RIP1-DsRed2 expression and 57.38% ± 6.12% with an increase in bGK-EGFP (Figure 7b). Stimulation of RIP1-DsRed2 by glucose was stronger than in mouse islets, while bGK-EGFP was stimulated by glucose to a similar extent [14]. βFLUOMETRI-transduced human islet microtissues are able to report on the metabolic status of the recipient animal similar to how mouse islets do.

### 2.5. In Vivo Measurement of Functional β-Cell Mass in Human Islet Microtissues during HFHSD in Rag1-KO Mice

When the NSG mice were put on a HFHSD we did not observe any effect on body weight or glucose tolerance. HFHSD induces β-cell insulin resistance in male C57Bl6/J (B6) mice. In βFLUOMETRI-transduced mouse islets transplanted to the ACE of B6 mice we observed that HFHSD led to a decrease in functional β-cell mass [14]. The reporter islet approach in that study allowed us to combine the assessment of robust parameters reflecting the metabolic state of the mice during diet intervention, such as intraperitoneal glucose tolerance test (IPGTT), intraperitoneal insulin tolerance test (IPITT), fasting blood glucose, and insulin levels, with data on β-cell function by monitoring reporter islets engrafted into the ACE.

Immune-deficient Rag1-KO mice have been used in high-fat diet (HFD) experiments and shown to increase their body weight and to develop impaired glucose tolerance [24]. These mice are also a suitable model for transplantation of human tissues [25]. To investigate the effect of HFHSD on human β-cell function in vivo, we transplanted βFLUOMETRI-transduced human islet microtissues (UNOS ID# AFFD319, 43 years old, female, White, BMI 34.28, HbA1c 4.6%) to the ACE of 2 months old male Rag1-KO mice. In total, 11 Rag1-KO mice were transplanted and fed a normal chow diet for 1 month. After a first in vivo imaging session (0 weeks of diet), the mice were divided into 2 experimental groups: 5 mice were fed a control diet and 6 mice were fed a HFHSD diet for 4 months. βFLUOMETRI fluorescence of the human microtissue grafts was imaged in vivo beginning 1 month after transplantation [22,23] and at 4, 8, and 12 weeks after start of HFHSD intervention with non-invasive imaging using laser scanning confocal microscopy. Human islet microtissues were imaged in anesthetized mice as 3D-stacks with 3 µm step-size before glucose stimulation. After the initial imaging and measuring fluorescence intensities of EGFP, DsRed2, and CFP by confocal microscopy, mice were allowed to recover from anesthesia, glucose was injected intraperitoneally and an IPGTT was performed. At 4 h post glucose injection, a second imaging was performed using the same setting as for the initial imaging to obtain the 4 h-image-set. These two image-sets were then used to calculate glucose stimulated promoter activation and functional β-cell mass.

**Figure 8:**
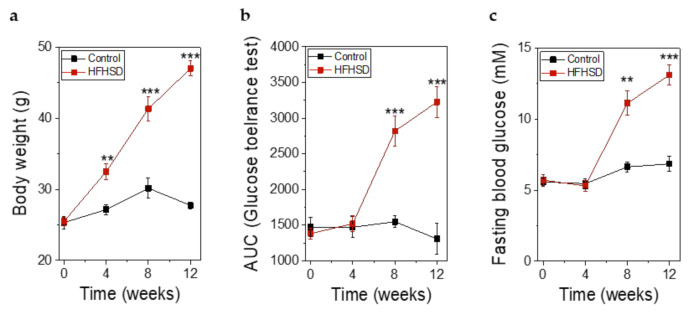
Metabolic parameters of Rag1-KO before, during, and after feeding of control diet or high-fat-high-sucrose diet (HFHSD) for 12 weeks. (**a**) Body weight before, during, and after feeding of control diet or HFHSD; (**b**) Glucose tolerance obtained by intraperitoneal glucose tolerance test (IPGTT) and depicted as the AUC of the IPGTT before, during, and after feeding of control diet or HFHSD; (**c**) Fasting blood glucose before, during, and after feeding of a control diet or HFHSD; (**a**–**c**) Data are expressed as means ± SEM, *n* = 5 for control diet and *n* = 6 for HFHSD, all parameters were obtained in mice after overnight fasting. The two-sided, unpaired *t*-test was used to determine statistical significance between different treatment groups. *** *p* < 0.001, ** *p* < 0.01.

**Figure 9:**
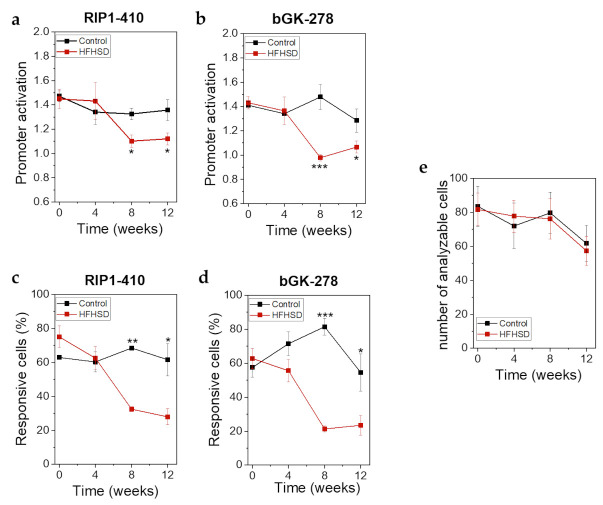
βFLUOMETRI revealed loss of functional β-cell mass in response to HFHSD in human islet microtissues transplanted into the ACE of Rag1-KO mice. (**a**) RIP1-410 promoter activation indicated by the βFLUOMETRI and obtained in vivo in transduced human islet microtissues transplanted into the ACE of Rag1-KO mice before, during, and after feeding of control diet or HFHSD; (**b**) bGK-278 promoter activation indicated by the βFLUOMETRI and obtained in vivo in human islet microtissues Rag1-KO mice before, during, and after feeding of control diet or HFHSD; (**c**) Percentage of glucose-responsive cells indicated by RIP1-410 of the βFLUOMETRI and obtained in vivo in transduced human islet microtissues transplanted into the ACE of Rag1-KO mice before, during, and after feeding of control diet or HFHSD; (**d**) Percentage of glucose-responsive cells indicated by bGK-278 of the βFLUOMETRI and obtained in vivo in transduced human islet microtissues transplanted into the ACE of Rag1-KO mice before, during, and after feeding of control diet or HFHSD; (**e**) Number of analyzable cells per transduced human islet microtissue transplanted into the ACE of Rag1-KO mice before, during, and after feeding of control diet or HFHSD; (**a**–**e**) Data are expressed as means ± SEM, *n* = 5 for control diet and *n* = 6 for HFHSD. The two-sided, unpaired *t*-test was used to determine statistical significance between different treatment groups. *** *p* < 0.001, ** *p* < 0.01, * *p* < 0.5.

Already after 4 weeks of HFHSD the body weight of the mice was significantly increased (Figure 8a). However, impaired glucose tolerance (Figure 8b) and increased fasting blood glucose (Figure 8c) did not show significant changes until 8 weeks of HFHSD. These parameters became more pronounced after 12 weeks of diet (Figure 8). Eight weeks of HFHSD led to a significant reduction in glucose-stimulated activities of RIP1-410 and bGK-278 in β-cells, which was also observed 12 weeks after start of diet intervention (Figure 9a,b). Because we intended to measure functional β-cell mass, we calculated the percentage of glucose-responsive β-cells (Figure 9c,d). RIP1-410 promoter activity demonstrated that 66.48 ± 0.83% of the β-cells were responsive to glucose stimulation at week 8 under control diet (Figure 9c), which decreased to 32.45 ± 1.17% in the HFHSD-fed group (Figure 9c). When bGK-278 activity was considered, the percentage of glucose-responsive β-cells changed within 8 weeks from 81.47 ± 4.88% for control diet to 21.27 ± 1.84% for HFHSD (Figure 9d). Figure 9e shows the number of fluorescent cells that could be analyzed over the 12-week diet period.

**Figure 10:**
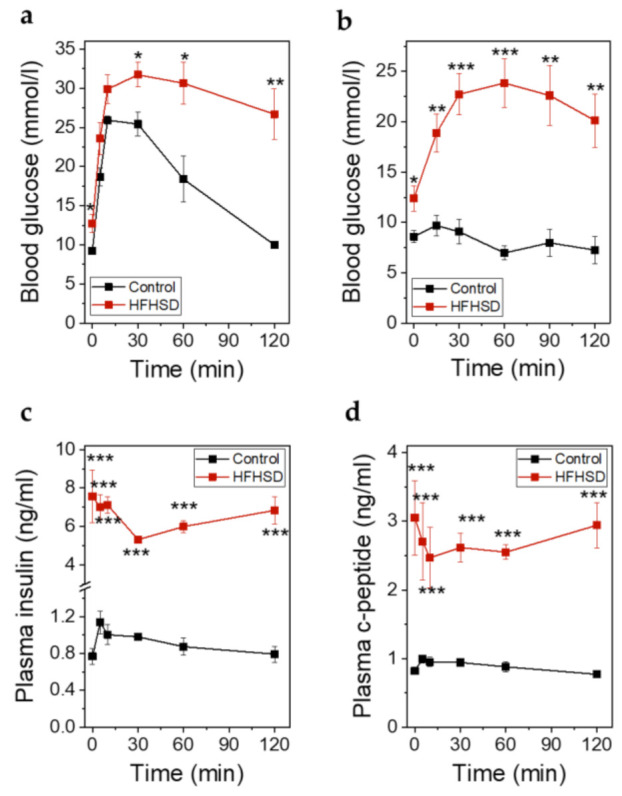
Metabolic parameters of Rag1-KO mice after feeding of control diet or HFHSD at the end of the study. (**a**) Blood glucose excursions during an IPGTT in mice fed control diet or HFHSD for 4 months; (**b**) Blood glucose excursions during an intraperitoneal insulin tolerance test (IPITT) in mice fed a control diet or HFHSD for 4 months; (**c**) Insulin secretion during an IPGTT in mice fed control diet or HFHSD for 4 months; (**d**) C-peptide secretion during an IPGTT in mice fed control diet or HFHSD for 4 months; (**a**–**d**): Data are expressed as means ± SEM, *n* = 5 for control diet and *n* = 6 for HFHSD, the results were obtained after 6 h of starvation. The two-sided, unpaired *t*-test was used to determine statistical significance between different treatment groups. *** *p* < 0.001, ** *p* < 0.01, * *p* < 0.5 for control diet vs. HFHSD.

At the end of the study when the animals had been fed the diets for 4 months, an IPITT was performed. At this timepoint the body weight was 30 ± 0.17 g in control diet-fed animals versus 48.28 ± 0.85 g in HFHSD-fed animals. IPGTT (Figure 10a) and IPITT (Figure 10b) were impaired in HFHSD-fed animals. Although the total amounts of secreted insulin (Figure 10c) and C-peptide (Figure 10d) during glucose stimulation in HFHSD-fed mice were increased, the levels of insulin were not sufficient to compensate for the hyperglycemia. The relative secretory response of β-cells, in terms of insulin and C-peptide release during the first minutes (up to 30 min) of the IPGTT, was significantly reduced in HFHSD-fed mice (Figure 10c,d).

In conclusion, human islet microtissues transplanted into the ACE of Rag1-KO mice fed a HFHSD also showed impaired β-cell function and reduced functional β-cell mass, comparable to B6-islets transplanted into B6 mice.

## 3. Discussion

The development of therapeutic approaches that can completely revert the course of β-cell dysfunction in T2D have been significantly slowed down by limitations of animal and in vitro models. Here we show how long-term in vitro exposure of human islet microtissues to glucotoxic and glucolipotoxic culture can reproducibly induce T2D relevant impairment in β-cell function with increased basal insulin secretion and decreased GSIS, the two hallmarks of β-cell dysfunction in T2D [15,16,17,18]. These models can now be applied for the discovery of mechanisms leading to loss of β-cell function under glucotoxicity/glucolipotoxicity as well as for the discovery of novel therapeutic agents with potential to prevent or reverse such dysfunction. Moreover, by establishing a method for highly-efficient viral transduction of fluorescent reporters and subsequent transplantation of the transduced islet microtissues, we were able to monitor human β-cell function and mass in vivo and concluded that loss of functional human β-cell mass also occurred in animal models of glucolipotoxicity, such as immune-deficient Rag1-KO mice fed HFHSD.

While the impact of high glucose concentrations (glucotoxicity) on the dysfunction and failure of β-cells in T2D is undisputed, the contribution of lipids (glucolipotoxicity) is more controversial and has recently been questioned [26]. Our results show that despite glucotoxicity being the main driver of β-cell dysfunction, lipotoxicity alone can increase basal insulin secretion, and therefore, may contribute to the consequent insulin resistance in peripheral tissues. Additionally, we observed that FFAs potentiate the deleterious effects of glucotoxicity on β-cell function, i.e., lower concentrations of glucose impair GSIS.

Another important outcome of our study is the possibility of performing highly efficient genetic manipulation through viral transduction in a model of standard human islets that displays robust and stable functionality, which in turn is scalable and compatible with disease modeling. Decreased insulin secretion, but not insulin content, was observed in the conditions with highest transduction efficiencies indicating a negative impact on protein processing and secretion machinery. Yet, stimulus-secretion coupling mechanisms were partially maintained, as a fold stimulation of insulin secretion higher than 6.7 was obtained in all groups.

Both sugar and fat overconsumption are believed to lead to obesity, insulin resistance, and subsequent development of T2D. In our previous work [14] we have shown that in rodents, β-cell function is impaired when specific dietary conditions (HFHSD) are brought onto a specific genetic background (B6). We showed that β-cell dysfunction occurs early during T2D progression and is provoked by the combination diet of high fat, which induces lipotoxicity, and sucrose, which triggers increased β-cell workload. Within 8 weeks of HFHSD, B6 mice became obese, developed impaired glucose tolerance and insulin resistance, and despite being hyperinsulinemic displayed non-compensatory insulin release, at least in part, due to reduced expression of syntaxin-1A [14].

In the current study, we show that human islet microtissues forms stable and well vascularized grafts following transplantation into ACE of Rag1-KO mice. Following HFHSD, the Rag1-KO mice became obese, developed impaired glucose tolerance and insulin resistance, and showed non-compensatory stimulated insulin release despite being hyperinsulinemic. Additionally, the mice developed impaired β-cell function and reduced functional β-cell mass, comparable to B6 islets in B6 mice [14]. The impairment in β-cell function could already be observed after 4 weeks of diet in mouse islets transplanted into B6 mice, whereas it took 8 weeks of HFHSD to observe similar dysfunction in human islet microtissues transplanted into Rag1-KO mice. In line with the results obtained by exposing islet microtissues to glucolipotoxicity in vitro, the results obtained in islet microtissues transplanted into the ACE of mice prove that human β-cells also become dysfunctional when exposed to glucolipotoxicity in an in vivo situation.

## 4. Materials and Methods

### 4.1. Reaggregated Human Islet Microtissue Production and Culture

InSphero 3D InSight human islet microtissues (InSphero AG, Schlieren, Switzerland) were produced by hanging-drop-based scaffold-free reaggregation of dispersed primary human islets obtained from Prodo Laboratories Inc. (Irvine, CA, USA). Consent was obtained from all next of kin, and there was no information on the identity of the donor for ethical and privacy reasons. For each production, between 10,000 and 20,000 islet equivalents (IEQs) were dispersed in dissociation solution (1992 µL 1X TrypLE Express, 12604013, Thermo Fisher Scientific, Waltham, MA, USA) plus 8 µL DNase I, 10 mg mL^−1^, to a final concentration of 40 µg mL^−1^ (10104159001, Sigma-Aldrich, St. Louis, MO, USA) by gentle pipetting at 37 °C. Remaining cell clumps were removed by filtering the cell suspension through a cell strainer (70 µm pore size). Then 2500 cells were seeded into each well of the InSphero Hanging Drop System and cultured for 4.5 days according to manufacturer’s instructions. The primary aggregates were then transferred to an Akura 96 plate to further mature for at least 2 more days before the start of the experiments. All experiments were performed 7–28 days after the start of the reaggregation. Islet microtissues were maintained in 3D InSight Human Islet Maintenance Medium (InSphero AG, Schlieren, Switzerland), renewed every 2–3 days.

### 4.2. Glucotoxic and Glucolipotoxic Cultures

To produce glucotoxic and glucolipotoxic medium, InSight Human Islet Maintenance Medium (InSphero AG, Schlieren, Switzerland) containing 5.5 mM glucose was supplemented with glucose to adjust the concentration to 8 mM, 11 mM, or 16.7 mM. For the production of the glucolipotoxic medium, separate solutions of sodium oleate (Sigma-Aldrich, Buchs, Switzerland, O7501) and sodium palmitate (P9767, Sigma-Aldrich, St. Louis, MO, USA) conjugated to FFA-free BSA (A8806, Sigma-Aldrich, St. Louis, MO, USA) in a 6:1 ratio (FFA:BSA) were prepared. These solutions were added to InSight Human Islet Maintenance Medium (with adjusted glucose concentrations) to reach a final concentration of 200 µM oleate and 100 µM palmitate. A solution with unconjugated FFA-free BSA was added to the culture medium of the healthy control group (5.5 mM glucose without FFA cocktail) to equilibrate the concentration of BSA (50 µM) in all treatment groups. Islet microtissues were cultured for 14 days in each corresponding medium, which was renewed every 2–3 days.

### 4.3. Adenoviral Transduction

The adenovirus encoding the β-cell fluorescent metabolic transcriptional-response indicator (βFLUOMETRI) was initially generated in [14] as follows: pENTR1A.RIP1.DsRed2/rbGK.EGFP/CMV.Cerulean was generated by inserting the RIP1.DsRed2/rbGK.EGFP cassette from pd2.RIP1.DsRed2/rbGK.EGFP into pENTR1A (Thermo Fisher Scientific, Waltham, MA, USA) and adding a CMV.Cerulean cassette downstream of the rbGK.EGFP cassette. The 3 individual expression cassettes are separated by transcription blocker sequences from the pd2EGFP-Promoter (Takara Bio, Kusatsu, Japan). All constructs were verified by DNA sequencing. The RIP1.DsRed2/rbGK.EGFP/CMV.Cerulean cassette was transferred into the promoterless adenovirus plasmid pAd/PL-DEST (Thermo Fisher Scientific, Waltham, MA, USA) by the Gateway technique. The ViraPower Adenoviral Expression System (Thermo Fisher Scientific, Waltham, MA, USA) was used to generate a replication-deficient adenovirus, which was used for transduction of cells and islets.

To transduce before reaggregation, dissociated islet cells were placed in Eppendorf tubes with medium and the corresponding number of viral particles, to a final volume of 1 mL. Tubes were closed with Parafilm, placed inside a Falcon tube, and shaken on a rotator at 37 °C for 1.5 h. Next tubes were centrifuged, supernatants were removed and, following resuspension, cells were seeded in InSphero Hanging Drop System (InSphero AG, Schlieren, Switzerland). Islet microtissue production continued as explained in Section 4.1.

To transduce during reaggregation, dissociated islet cells were mixed at the required concentration with the corresponding number of viral particles in medium. The cell suspensions containing the adenovirus were seeded in InSphero Hanging Drop System and islet microtissue production continued as explained in Section 4.1, with the inclusion of two washing steps with InSight Human Islet Maintenance Medium after transferring the islet microtissues to an Akura 96 plate.

To transduce after reaggregation, islet microtissues were produced as in Section 4.1. The Akura plates containing islet microtissues were centrifuged and medium was removed. 70 µL of InSight Human Islet Maintenance Medium containing the corresponding number of viral particles were added to each islet microtissue and the plates were placed in an incubator at 37 °C for 2 h. Then, 2 wash steps with InSight Human Islet Maintenance Medium were performed.

### 4.4. In Vitro Insulin Secretion Assay and Quantification of Insulin, ATP, and Caspase 3/7 Activity

To prepare the islet microtissues for static GSIS, culture medium corresponding to the last 48–72 h in culture was removed and stored for the analysis of chronic insulin secretion. Islet microtissues were washed twice with 70 µL Krebs-Ringer Hepes-bicarbonate Buffer (KRHB; 131 mM NaCl, 4.8 mM KCl, 1.3 mM CaCl2, 25 mM Hepes, 1.2  mMm KH2PO4, 1.2 mM MgSO4, 0.5% BSA) containing 2.8 mM glucose and equilibrated for 1 h in the same solution. GSIS was performed in the Akura 96 plate in 50 µL KRHB containing different glucose concentrations for 2 h. The supernatant was collected for ELISA analysis. After GSIS, the microtissues were lysed to analyze total ATP content using CellTiter-Glo Luminescent Cell Viability Assay (Promega, Madison, WI, USA with protease inhibitor cocktail G6521 from the same company) and a microplate reader (Infinite M1000, TECAN, Männedorf, Switzerland). The lysates were then used for assessment of total insulin content. Alternatively, the microtissues were lysed to analyze caspase 3/7 activity using Caspase-Glo^®^ 3/7 Assay System (G8091, Promega, Madison, WI, USA). After proper dilutions in KRHB were performed, total and secreted insulin was quantified using Stellux Chemi Human Insulin ELISA (80-INSHU-CH10, Alpco, Salem, NH, USA). The obtained insulin and ATP values are shown unnormalized, expressed as ng of insulin or pmol of ATP per islet microtissue.

### 4.5. Immunofluorescence Confocal Microscopy and Image Analysis for Quantification of Adenoviral Transduction Efficiency and Homogeneity 

Islet microtissues were treated with nuclear dye DRAQ5 for 2 h prior to fixation. Then, microtissues were fixed for 30 min in 2% paraformaldehyde (PFA) at RT. PFA was removed, microtissues were washed twice with PBS and kept in PBS with 0.05% azide until imaging.

Image acquisition and analysis was performed by Visikol (Hampton, NJ, USA). DRAQ5 treated microtissues were optically cleared with the Visikol^®^ HISTO™ system (Visikol, NJ, Hampton, USA) and were imaged by 4-channel high content confocal microscopy using a CX7 LZR (Thermo Fisher Scientific, Waltham, MA, USA) in a 384-well glass bottom plates (CellCarrier-384 Ultra Microplates, PerkinElmer, Waltham, MA, USA, 6057300) with 5 µm Z-steps for the following fluorophores: Cerulean (Excitation/Emission max 435/475 nm), eGFP (Excitation/Emission max 488/510 nm), DsRed2 (Excitation/Emission max 556/584 nm), and DRAQ5 (Excitation/Emission max 647/681 nm).

Nuclei were detected, segmented, and counted using a customized CellProfiler 2.2.0 (www.cellprofiler.org, Cambridge, MA, USA) pipeline. Coordinates (X,Y,Z) of nuclei were used to extract intensity values of CFP, EGFP, and DsRed2 channels for each nucleus. Transduction efficiency was calculated as a percentage by dividing the number of CFP positive cells by the total number of DRAQ5 nuclei in each islet microtissue. Transduction homogeneity was evaluated by calculating the transduction efficiency in three predefined regions of each islet. These regions were defined as follows: the radius of each nuclear point to the manually selected center-point of the spheroid was calculated. Radii distances were normalized by dividing by the maximum radius obtained for the microtissue. Nuclei were grouped into three bins based on normalized radius from center: bin 1, inner core (r0 = 0–0.333), bin 2, middle third (r0 = 0.334–0.666) and bin 3, outer third (0.666–1.0).

### 4.6. Immunofluorescence Confocal Microscopy and Image Analysis for Quantification of Proliferation and Apoptosis 

Islet microtissues were washed twice with PBS and fixed for 30 min in 2% PFA at RT. PFA was removed, microtissues were washed twice with PBS and kept in PBS with 0.5% azide until immunofluorescent staining.

Staining, image acquisition and analysis was performed by Visikol (Hampton, NJ, USA). Islet microtissues were permeabilized with 0.2% Triton X-100 in PBS, followed by incubation in Penetration Buffer containing 0.2% Triton X-100 and 20% DMSO for 1 h each. The tissues were then blocked with a 6% donkey serum in PBS buffer solution for 1 h at 37 °C. The primary antibodies used for immunolabeling were Rabbit Anti-Ki67 (RB-1510, Thermo Fisher Scientific, Waltham, MA, USA) at a 1:200 dilution, Guinea Pig Anti-PDX1 (ab47308, Abcam, Cambridge, UK) at a 1:100 dilution, and Mouse Anti-Caspase3 (MA5-11516, Thermo Fisher Scientific, Waltham, MA, USA) at a 1:150 dilution in Antibody Buffer containing 3% donkey serum in PBS buffer. Tissues were washed 3 times between primary and secondary labeling steps with washing buffer composed of 0.2% Tween in PBS. The secondary antibody dilutions used were 1:200 for Goat Anti-Guinea Pig Alexa 488, Anti-Rabbit Alexa 568, and Anti-Mouse Alexa 674, along with DAPI at a 1:1000 dilution (all from Thermo Fisher Scientific, Waltham, MA, USA). Tissues were washed and transferred to the film-bottomed Akura™ 384 plate (InSphero AG, Switzerland), and cleared with Visikol^®^ HISTO™ system (Visikol, NJ, Hampton, USA).

High content imaging of islet microtissues was performed using a CX7-LZR (Thermo Fisher Scientific, Waltham, MA, USA) platform. Twenty optical sections were acquired at 5 µm intervals for each well/channel with 20X magnification. Image stacks of the individual microtissues were quantified using customized CellProfiler 2.2.0 (www.cellprofiler.org, Cambridge, MA, USA) pipelines.

### 4.7. Animals and Diet

Male NOD.Cg-Prkdc^scid^ Il2rg^tm1Wjl^/SzJ (NSG) and male B6.129S7-Rag1^tm1Mom^/J (Rag1-KO) mice were purchased at 2 months of age from The Jackson Laboratory (Bar Harbor, ME, USA). After delivery, mice were left to adapt to the animal core facility for 1 week before the start of the experiment. All mice were group-housed at a 12/12 h dark-light-cycle with free access to food and water. If not otherwise indicated, the mice received a normal chow diet (R70, Lantmännen, Klimstad, Sweden). Starting at month 3, NSG and Rag1-KO mice received a HFHSD, consisting of a solid HFD (60% kcal from fat, TD.06414, Envigo, Venray, Netherlands) and 32% sucrose solved in tap water, for 16 weeks. A control diet was given to the control group: Managed formulation purified ingredient diet (#5P76, LabDiet, St. Louis, MO, USA) and tap water. The control diet given to the control group differs from the R70 diet all animals received before the diet intervention. Consequently, all the animals underwent a change of diet. All experiments were performed in accordance with the Karolinska Institutet’s guidelines for the care and use of animals in research and were approved by the institute’s Animal Ethics Committee.

### 4.8. Transplantation of Pancreatic Islets into the ACE

Islets microtissues were transplanted into the ACE of 2 months old NSG and Rag1-KO mice using a technique described previously [22]. Briefly, under anesthesia, islets of Langerhans were transplanted into the ACE using a glass cannula after generating a puncture in the cornea with a 27-gauge needle. Great care was taken to avoid bleeding and damage to the iris. Mice were injected subcutaneously with Temgesic (0.1 mL/kg, RB Pharmaceuticals Limited, Berkshire, UK) for postoperative analgesia.

### 4.9. In Vivo Imaging of Intraocular Islet Grafts and Image Analysis

Islet grafts were imaged in vivo beginning 1 month after transplantation and at 4, 8 and 12 weeks after start of diet intervention. An upright laser scanning confocal microscope (Leica TCSSP5, LEICA Microsystems, Wetzlar, Germany) equipped with a long-distance water-dipping objective (Leica HXC-APO10×/0.30 NA) and a custom-built stereotaxic head holder allowing positioning of the mouse eye containing the engrafted islets toward the objective was used. Viscotears (Théa Nordic AB, Örebro, Sweden) was used as an immersion liquid between the eye and the objective, and isoflurane was used to anesthetize the mice during in vivo imaging. Islets were imaged as 3D-stacks with 3 µm step-size. After imaging, the mice were allowed to recover from anesthesia and an IPGTT was performed. At 4 h post glucose injection, a second imaging was performed using the same setting as described above to obtain the 4 h-image set.

β-cell fluorescent metabolic transcriptional response indicator (βFLUOMETRI): Cerulean fluorescence was excited at 405 nm and detected at 460 to 490 nm. EGFP fluorescence was excited at 488 nm and detected at 505 to 535 nm. DsRed2 fluorescence was excited at 561 nm and detected at 580 to 650 nm. Backscatter signal from the 561 nm excitation was collected at 555 to 565 nm. Channel crosstalk was avoided by using in-between-lines sequential imaging combining CFP (Cerulean), backscatter, and DsRed2 signal and separating the EGFP signal.

Image analysis for in vitro experiments was performed as described in [14] using ImageJ. Average fluorescence intensities for each cell were determined for t = 60 min (start) and t = 240 min. Images derived from in vivo imaging were analyzed using Leica LAS software (Leica Microsystems). For each analyzed cell, fluorescence intensity for all 3 fluorescent dyes (CFP, EGFP, DsRed2) was determined before glucose stimulation and 4 h after glucose stimulation. To calculate the change in promoter activity after glucose stimulation as readout for β-cell functionality, fluorescence intensities for each cell were determined at the beginning of the experiment and 4 h after glucose stimulation. Cells were identified using the DsRed2-fluorescence signal thereby ensuring the analysis of β-cells. Promoter activation was calculated as follows: ((EGFP/DsRed2_4h_-Background_4h_)/(CFP_4h_-Background_4h_))/((EGFP/DsRed2_start_-Background_start_)/(CFP_start_-Background_start_)). To determine functional β-cell mass as a percentage of glucose responsive cells, cells with a promoter activation >1.15 were considered responsive.

Imaging of vascularization: for blood vessel imaging mice were injected intravenously with 100 μL TRITC-labelled dextran (2.000.000 MW, 5 mg/mL, Thermo Fisher Scientific, Waltham, MA, USA). TRITC fluorescence was excited at 555 nm and detected at 580 to 620 nm. Backscatter signal from the 633 nm excitation was collected at 630 to 640 nm.

### 4.10. IPGTT

To determine glucose tolerance, blood glucose levels were measured in mice fasted for 6 h (or together with the imaging after overnight fasting) at basal state (0 min), 5, 10, 30, 60, and 120 min after intraperitoneal glucose injection (2 g/kg body weight, dissolved in PBS). The results were depicted as area under the curve (AUC) of the IPGTT. Glucose concentrations were measured using the Accu-Chek Aviva monitoring system (Roche, Basel, Switzerland).

### 4.11. IPITT

To measure whole body insulin resistance, the IPITT was performed determining glucose blood levels after an insulin challenge. Blood glucose concentration was measured in mice fasted for 6 h (basal state, 0 min). Then, mice were intraperitoneally injected with insulin (0.25 U/kg body weight, diluted in PBS, Novo Nordisk, Bagsværd, Denmark) followed by intraperitoneal glucose administration (1 g/kg body weight) and blood glucose concentrations were determined at 15, 30, 60, 90, and 120 min after glucose injection. The results were depicted as AUC of the IPITT.

### 4.12. Body Weight and Fasting Blood Glucose

Body weight and fasting blood glucose were measured after 6 h fasting time or overnight fasting.

### 4.13. Plasma Biology

Blood samples were collected at different time points during the IPGTT, centrifuged at 5000× *g* during 30 min for plasma obtention and preserved at −20 °C until use. Ultrasensitive mouse enzyme-linked immunosorbent assay (ELISA) kits (CrystalChem, Elk Grove Village, IL, USA) were used to analyze plasma insulin and C-Peptide.

### 4.14. Statistics

GraphPad Prism 9 (GraphPad, San Diego, CA, USA), Origin 2015 64 Bit (OriginLab, Northampton, MA, USA) and Microsoft Office Excel (Microsoft, Redmont, WA, USA) were used for statistical analysis.

## 5. Conclusions

In summary, our data reveal that human islet microtissues:

1. Are amenable to disease modeling with glucotoxicity and glucolipotoxicity, which induced T2D-like β-cell dysfunction.
2. Can be successfully transduced with adenoviruses with high efficiency.
3. Survive, engraft, and vascularize when transplanted into the ACE of immune-deficient mice.
4. Report on the state of β-cells and functional β-cell mass during diet interventions in mice when equipped with fluorescent biosensors.
5. Exhibit a loss in β-cell functionality when exposed to glucolipotoxic conditions in vivo.

## Figures and Tables

**Figure A1:**
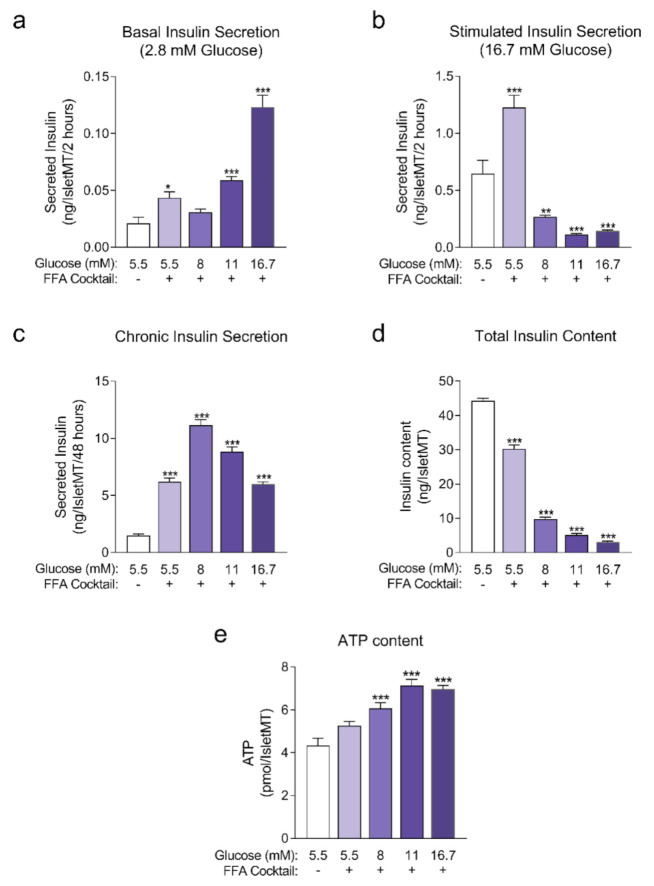
(**a**) Basal insulin secretion at 2.8 mM glucose at the end of 14 days of incubation (*n* = 6); (**b**) Stimulated insulin secretion at 16.7 mM glucose at the end of 14 days of incubation (*n* = 6); (**c**) Chronic insulin secretion to the culture media corresponding to the last 48 h of incubation (*n* = 12); (**d**) Intracellular insulin content of islet microtissues after 14 days of incubation (*n* = 6). (**e**) Intracellular ATP content of islet microtissues (*n* = 5–6). Outliers were detected with ROUT’s test (Q = 5%). Statistical analysis was performed with one-way ANOVA. Dunnett’s multiple comparisons tests against the 5.5 mM glucose (without FFA) control of each condition are shown: *** *p* < 0.001, ** *p* < 0.01, * *p* < 0.5.

**Figure A2:**
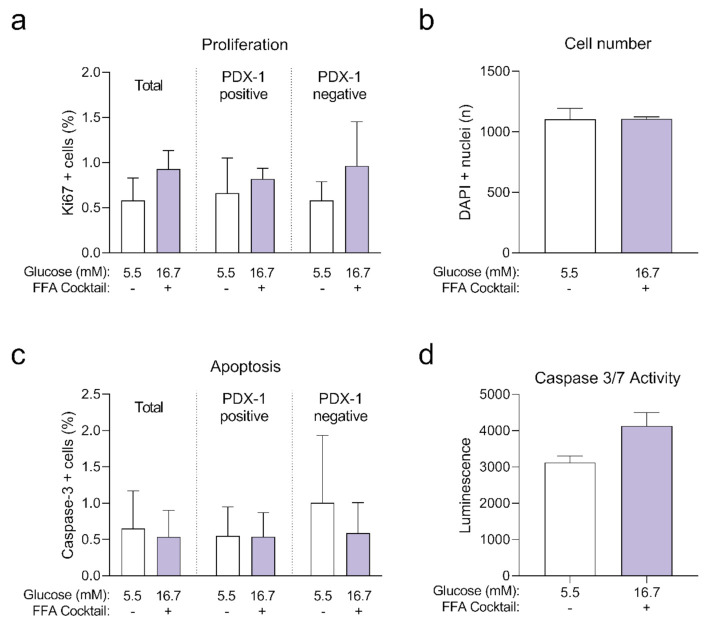
Positive cells in the total, PDX-1 positive and PDX-1 negative populations at the end of 7 days of incubation (*n* = 3 donors with *n* = 6 islet microtissues per donor); (**b**) Total number of cells analyzed represented as DAPI positive nuclei count. (**c**) Apoptosis expressed as the percentage of cleaved caspase-3 positive cells in the total, PDX-1 positive and PDX-1 negative populations at the end of 7 days of incubation (*n* = 3 donors with *n* = 6 islet microtissues per donor); (**d**) Caspase 3/7 activity was analyzed with a lytic assay at the end of 7 days of incubation (*n* = 3 donors with *n* = 6 islet microtissues per donor). Outliers were detected with ROUT’s test (Q = 5%). Statistical analysis was performed with Student’s *t*-test.

**Figure A3:**
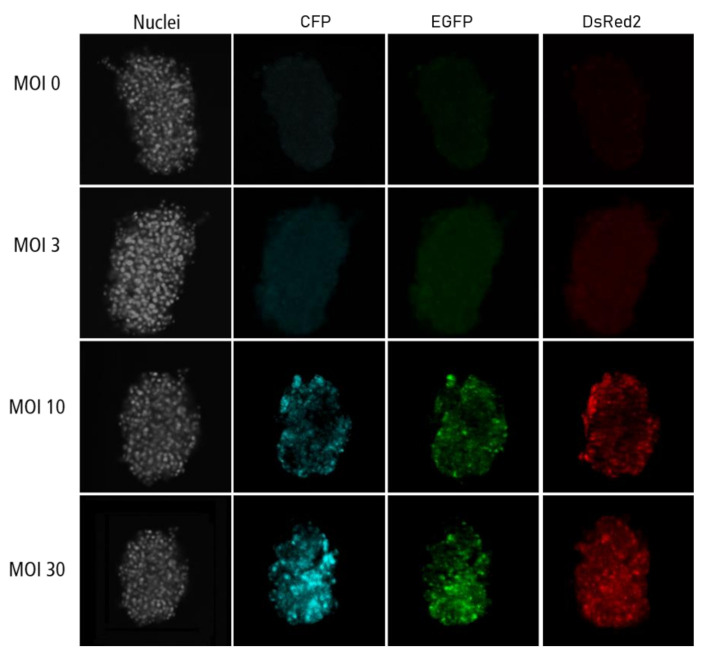
Confocal microscopy images for each channel of islet microtissues transduced before reaggregation.

**Figure A4:**
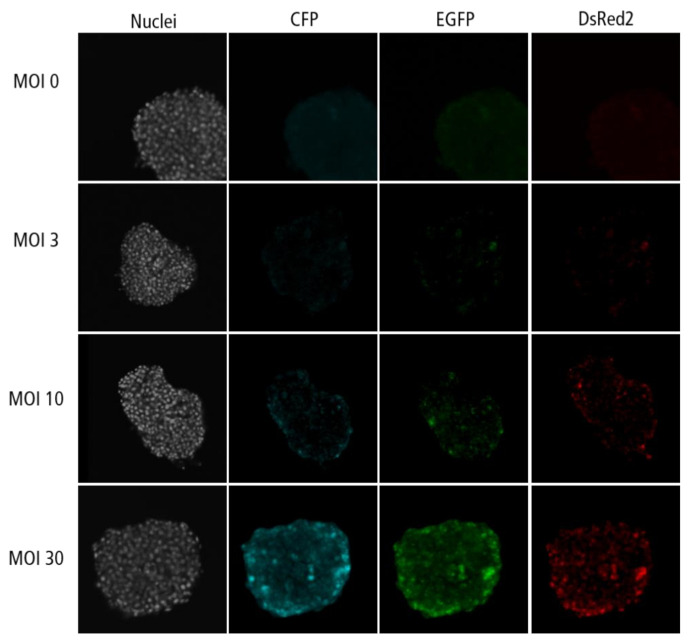
Confocal microscopy images for each channel of islet microtissues transduced during reaggregation.

**Figure A5:**
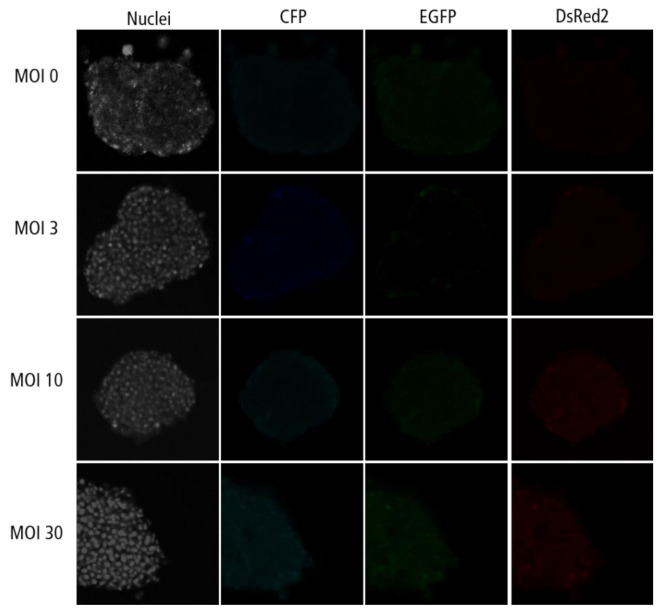
Confocal microscopy images for each channel of islet microtissues transduced after reaggregation.

## Data Availability

All data are available on request from the authors.

## Abbreviations

T2D	Type 2 diabetes mellitus
BMI	Body mass index
HFHSD	High-fat-high-sucrose diet
ACE	Anterior chamber of the eye
UNOS ID	United Network for Organ Sharing Identifier
HbA1c	Glycated hemoglobin
FFA	Free fatty acid
GSIS	Glucose-stimulated insulin secretion
CFP	Cerulean fluorescent protein
EGFP	Enhanced green fluorescent protein
CMV	Cytomegalovirus
RIP	Rat Insulin 1 promoter
bGK	β-cell–active glucokinase gene promoter
MOI	Multiplicity of infection
IPGTT	Intraperitoneal glucose tolerance test
IPITT	Intraperitoneal insulin tolerance test
IPPTT	Intraperitoneal pyruvate tolerance test
HFD	High-fat diet
KRHB	Krebs-Ringer Hepes-bicarbonate buffer
PFA	Paraformaldehyde
RT	Room temperature

## Appendix A

Human islet microtissues from a different islet donor (UNOS ID# AEAI043, 27 years old, female, Hispanic, BMI 27.77, HbA1c 5.0%) were exposed for 14 days to increasing concentrations of glucose in the culture medium, with the addition of a mix of saturated and unsaturated FFAs.

## Appendix B

Human islet microtissues from three different islet donors (Donor 1: UNOS ID# AEL5345, 48 years old, male, Hispanic, BMI 32.38, HbA1c 5.6%; Donor 2: UNOS ID# AFCS437, 46 years old, male, Hispanic, BMI 33.2, HbA1c 5.4%; Donor 3: UNOS ID# AGIJ272, 64 years old, male, Caucasian, BMI 25.5, HbA1c 5.4%) were exposed for 7 days to 16.7 mM glucose in the culture medium with the addition of a mix of saturated and unsaturated FFAs, and compared to a healthy control group cultured at 5.5 mM glucose without FFAs. Then, islets were fixed and stained for Ki67 and cleaved caspase-3 to quantify proliferation (Figure A2a) and apoptosis respectively (Figure A2b) with confocal 3D microscopy. No significant differences were observed between both groups.

Human islet microtissues from three different islet donors (Donor 1: UNOS ID# AEL5345, 48 years old, male, Hispanic, BMI 32.38, HbA1c 5.6%; Donor 2: UNOS ID# AFCS437, 46 years old, male, Hispanic, BMI 33.2, HbA1c 5.4%; Donor 3: UNOS ID# AGEM175, 34 years old, male, Hispanic, BMI 28.65, HbA1c 5.2%) were exposed for 7 days to 16.7 mM glucose in the culture medium with the addition of a mix of saturated and unsaturated FFAs, and compared to a healthy control group cultured at 5.5 mM glucose without FFAs. Then, islets were lysed and caspase3/7 activity was measured (Figure A2c). No significant differences were observed between both groups.
